# Molecular Transmission Clustering and Pretreatment Drug Resistance Among Men Infected With HIV-1 Who Have Sex With Men in Baotou, China: A Cross-Sectional Subgroup Analysis

**DOI:** 10.2196/82208

**Published:** 2026-11-06

**Authors:** Hanping Li, Yu Liu, Hong Yang, Jiao Wu, Ziwei Liu, Baobao Bai, Yongjian Liu, Lei Jia, Jingwan Han, Bohan Zhang, Xiaolin Wang, Xiangchun Li, Lin Li

**Affiliations:** 1 State Key Laboratory of Pathogen and Biosecurity, Academy of Military Medical Sciences, 20 Dongda Street, Fengtai District, Beijing, 100071, China, +86-13460359811; 2 School of Public Health, Inner Mongolia Medical University, Hohhot, China; 3 Department of HIV/AIDS Control and Prevention, Inner Mongolia Autonomous Region Center for Disease Control and Prevention, Hohhot, China; 4 School of Public Health, Hebei Medical University, Shijiazhuang, China

**Keywords:** HIV‐1, phylogenetic analysis, genetic clustering, molecular transmission network, pretreatment drug resistance

## Abstract

**Background:**

In the Inner Mongolia Autonomous Region, men who have sex with men (MSM) have become a high-risk group for HIV transmission due to their complex, secretive sexual relationships, posing challenges to HIV-1 prevention.

**Objective:**

We analyzed factors associated with molecular transmission clustering and pretreatment drug resistance (PDR) among newly reported HIV-1–infected MSM in Baotou, China, and to provide locally relevant evidence for targeted public health interventions.

**Methods:**

We conducted a subgroup cross-sectional molecular epidemiological analysis of all newly reported HIV-1 cases attributed to homosexual transmission in Baotou between January 2022 and December 2024. The sample comprised 213 treatment-naïve MSM, from whom plasma samples were obtained. A 1.3-kb fragment of the HIV-1 pol gene was amplified and sequenced using next-generation sequencing. Inclusion criteria were a newly reported HIV-1 infection, no antiretroviral therapy at the time of this writing, and self-reported same-sex sexual behavior. Primary outcomes were clustering in a genetic distance–based molecular transmission network and the presence of PDR according to the Stanford HIV Drug Resistance Database.

**Results:**

In total, 176 pol gene sequences were successfully obtained, with circulating recombinant form (CRF)–07_BC being the most prevalent subtype, accounting for 56.82% of the total sequences. The molecular transmission network was constructed using a pairwise genetic distance of 1.30%, with 52 of 176 (29.55%) sequences forming 19 clusters within the network. The network exhibited significant assortative mixing by age (ra=0.41; *P*=.001), marital status (ra=0.408; *P*=.002), and educational attainment (ra=0.385; *P*=.001), rather than random mixing. Multivariate logistic regression analysis revealed that CRF07_BC subtype–infected individuals had a significantly higher risk of clustering (AOR 4.98; 95% CI 1.92‐12.94; *P*<.01). Compared to never-married individuals, divorced or widowed individuals were less likely to have clustered cases (AOR 0.27; 95% CI 0.08‐0.92; *P*<.05). The prevalence of PDR was 6.57% (n=14). Nonnucleoside reverse transcriptase inhibitor resistance was the most common (n=8, 3.76%), followed by protease inhibitor resistance (n=5, 2.35%), and nucleoside reverse transcriptase inhibitor resistance (n=3, 1.14%). Drug-resistant strains were more frequently detected among individuals infected with the CRF07_BC subtype (*χ*^2^_8_=90.44; *P<*.001).

**Conclusions:**

Unlike previous studies primarily focused on high-burden southeastern coastal regions of China, we investigated the molecular transmission characteristics of HIV-1 among MSM in the Inner Mongolia Autonomous Region. Our findings demonstrate that transmission clustering and drug resistance patterns previously observed are also present in northern border areas. This study integrated HIV-1 molecular transmission network analysis with next-generation sequencing technologies to identify key populations involved in local HIV transmission, thereby supporting targeted public health interventions. In addition, surveillance of PDR among high-risk populations may facilitate the development of evidence-based antiretroviral treatment strategies and improve the efficient allocation of limited health care resources.

## Introduction

AIDS is a chronic infectious disease caused by HIV. AIDS continues to pose substantial challenges to public health, demographic stability, and socioeconomic development worldwide. The US Centers for Disease Control and Prevention (CDC) reports that in 2023, over 80% of those diagnosed with HIV were men, with 66% of those cases linked to men who have sex with men (MSM) [1]. A nationwide study showed that in some regions of China, 21.4%-23.4% of new AIDS cases were related to HIV transmission among MSM [2,3]. The HIV-1 prevalence rate among MSM in China rose from 5.7% in 2010 to 23.0% in 2019 [4]. As a relatively small group with complex social relationships, some MSM may simultaneously engage in both receptive and insertive sexual behavior, which accelerates HIV transmission. Therefore, given the critical role that MSM play in HIV-1 prevention and management efforts, studying their molecular epidemiological characteristics is of vital importance.

Due to underreporting in MSM, the actual proportion of infections transmitted through MSM may be underestimated. Socioepidemiological investigations can provide some information on HIV-1 transmission patterns, but the secrecy of MSM sexual behaviors limits the reliability of the information obtained on HIV transmission [5]. Interventions targeting individual high-risk behaviors have had limited effectiveness in eliminating HIV/AIDS. Consequently, research focus has shifted to the role of networks in HIV transmission, including sexual transmission networks, social networks, and molecular transmission networks [6]. With continuous advances in genomic sequencing technologies and the ongoing accumulation of data on HIV drug resistance, researchers have increasingly focused on molecular transmission networks constructed from viral sequence data. These networks offer unique advantages. They can trace viral transmission pathways and identify clusters of drug-resistant strains, pinpoint risk factors for HIV transmission, and reveal potential transmission routes within transmission chains, thereby facilitating targeted interventions. The use of molecular transmission networks to identify clusters of high-risk individuals supplements the incomplete information obtained solely through studying social and sexual behavior patterns among people living with HIV [7,8]. By using these insights, public health measures can more effectively curb viral transmission and reduce infection rates [9-11].

Antiretroviral therapy (ART) remains the best treatment option for HIV/AIDS [12]. It effectively suppresses viral replication, reduces mortality, and improves patients’ quality of life [13,14]. With the expansion of ART coverage, most patients achieve viral suppression and maintain a certain level of immune function following antiviral treatment. However, with the widespread use of antiviral drugs, the virus continues to undergo genetic mutations under selective pressure, leading to reduced drug sensitivity and the development of drug resistance, which in turn results in treatment failure. Some drug-resistant mutant strains may spread from treatment-failed patients to untreated individuals, and certain mutant strains with transmission advantages may spread widely in the population, leading to increased proportions of transmission-associated drug resistance and pretreatment drug resistance (PDR) [15-17]. PDR refers to drug resistance detected in individuals with no prior history of ART exposure. PDR may reduce the efficacy of ART, increase mortality, and lead to secondary transmission of HIV [16,18]. Therefore, assessing PDR levels helps to better understand the current status of HIV-1 drug resistance in specific regions and provides a foundation for formulating alternative treatment strategies.

In China, individual ART regimens are primarily determined by national standard first-line and second-line treatment strategies [19], and people living with HIV should initiate antiviral treatment as early as possible, within 30 days of diagnosis. Typically, individual HIV genotypic resistance testing is required before starting ART. Failure to identify drug-resistant strains in a timely manner may facilitate onward viral transmission. Since 2011, the prevalence of PDR in China has shown an upward trend [20]. Data from a multicenter observational study indicate that the overall prevalence of PDR in China was 7.40% in 2022 [19]. Previous studies have shown that while several common mutations account for the transmission of most subtypes and cases of high-level resistance, the prevalence of PDR among HIV-1-infected MSM varies geographically. For example, the prevalence of PDR was 12.72% in Guiyang [21], and in Chongqing it was 5.60% [22].

Inner Mongolia Autonomous Region is located in the northern frontier of China, and Baotou is situated in the central-western part of the region. Between 2022 and 2024, 99.36% of newly reported cases in Baotou were sexually transmitted infections, with MSM accounting for 65.14% of these cases. Although homosexual transmission is predominant in the city, research on the transmission patterns among MSM remains limited. Therefore, the primary objectives of this study were (1) to characterize the HIV-1 molecular transmission network and identify factors associated with phylogenetic clustering among newly reported MSM in Baotou; (2) to assess the prevalence and patterns of PDR in this population; and (3) to provide locally relevant evidence for targeted public health interventions. An analysis of representative cities is not only crucial for refining and evaluating the effectiveness of HIV-1 prevention and control strategies in the Inner Mongolia Autonomous Region but also provides valuable insights for other cities. This approach facilitates a deeper understanding of HIV-1 transmission risks at the local level and enables the rational distribution of limited public health resources to achieve targeted interventions against HIV/AIDS.

## Methods

### Study Design

This study is a subgroup analysis derived from a broader HIV-1 molecular surveillance project conducted in Baotou, Inner Mongolia Autonomous Region, between January 2022 and December 2024 [23]. This subgroup analysis was preplanned to address the research questions specific to the MSM population, which accounts for the highest proportion of newly reported HIV-1 cases in the region.

### Inclusion and Exclusion Criteria

Inclusion criteria were (1) newly reported HIV-1 infection, (2) no prior exposure to ART, and (3) men who self-reported having sex with men. Exclusion criteria were (1) individuals with a prior history of ART exposure, (2) individuals who declined to provide written informed consent or who withdrew consent during the study period, and (3) cases where plasma samples were insufficient in volume (<200 μL).

### Participant Characteristics

A total of 213 ART-naïve MSM were enrolled in this study. Their median age was 33 (IQR 26‐45) years. Additional demographic and clinical characteristics of the 176 participants with successfully sequenced samples are summarized in Table 1.

**Table 1.** Demographics and HIV-1 subtypes in men who have sex with men (MSM)–infected individuals.

Variables	Participants (N=176)	Subtype			HIV-1 subtype B (n=6)	Other^a^ (n=9)	*P* value*^b^*
		CRF^c^01_AE (n=51)	CRF07_BC (n=100)	CRF55_01B (n=10)			
Reported (year), n (%)							.12
2022	63 (35.80)	19 (30.16)	35 (55.56)	4 (6.35)	2 (3.17)	3 (4.76)	
2023	64 (36.36)	14 (21.88)	39 (60.94)	6 (9.38)	2 (3.13)	3 (4.69)	
2024	49 (27.84)	18 (36.73)	26 (53.06)	0 (0)	2 (4.08)	3 (6.12)	
Ethnicity, n (%)							.56
Han Chinese	162 (92.05)	47 (29.01)	91 (56.17)	9 (5.56)	6 (3.7)	9 (5.56)	
Mongol	12 (6.82)	4 (33.33)	8 (66.67)	0 (0)	0 (0)	0 (0)	
Hui	2 (1.14)	0 (0)	1 (50.00)	1 (50.00)	0 (0)	0 (0)	
Age (years), n (%)							.49
<30	59 (33.52)	18 (30.51)	31 (52.54)	4 (6.78)	3 (5.08)	3 (5.08)	
30‐50	91 (51.70)	26 (28.57)	53 (58.24)	5 (5.49)	2 (2.20)	5 (5.49)	
>50	26 (14.77)	7 (26.92)	16 (61.54)	1 (3.85)	1 (3.85)	1 (3.85)	
Marital status, n (%)							.05
Never married	117 (66.48)	35 (29.91)	65 (55.56)	7(5.98)	5 (4.27)	5 (4.27)	
Married	34 (19.32)	7 (20.59)	22 (64.71)	3 (8.82)	0 (0)	2 (5.88)	
Divorced/widowed	25 (14.20)	9 (36.00)	13 (52.00)	0 (0)	1 (4.00)	2 (8.00)	
Education level, n (%)							.07
Primary school and illiteracy	10 (5.68)	6 (60.00)	4 (40.00)	0 (0)	0 (0)	0 (0)	
Junior middle school	26 (14.77)	10 (38.46)	11 (42.31)	1 (3.85)	0 (0)	4 (15.38)	
Senior middle school and above	140 (79.55)	35 (25.00)	85 (60.71)	6 (4.29)	9 (6.43)	5 (3.57)	
CD4+^d^ T lymphocytes, n (%)							.04
<200	43 (24.43)	15 (34.88)	19 (44.19)	6 (13.95)	2 (4.65)	1 (2.33)	
200‐500	91 (51.70)	24 (26.37)	55 (60.44)	3 (3.30)	2 (2.20)	7 (7.69)	
>500	42 (23.86)	12 (28.57)	26 (61.90)	1 (2.38)	2 (4.76)	1 (2.38)	
Registered residence, n (%)							.51
Baotou	155 (88.07)	46 (29.68)	86 (55.48)	10 (6.45)	5 (3.23)	8 (5.16)	
Other regions in the Inner Mongolia Autonomous Region	5 (2.84)	0 (0)	4 (80.00)	0 (0)	0 (0)	1 (20.00)	
Other provinces	16 (9.09)	5 (31.25)	10 (62.5)	0 (0)	1 (6.25)	0 (0)	

a Other includes CRF67_01B subtype (n=3), CRF59_01B subtype (n=1), CRF08_BC subtype (n=1) and unique recombination form (n=4).

b Corrected chi-square test was used for reporting year and Fisher exact probability method was used for the remaining variables.

c CRF: circulating recombinant form.

d CD4+: cluster of differentiation 4.

### Sampling Procedure

From January 2022 to December 2024, all newly reported cases of HIV-1 infection among MSM in Baotou were continuously recruited through the local CDC. Blood samples were collected after confirmation of infection and prior to initiation of ART. Plasma samples were separated and stored at −80 °C immediately after collection. This continuous enrollment approach resulted in a sampling coverage rate of 91.42% (n=213) for all newly reported MSM in the city during the study period.

### Sample Size Determination

The sample size was determined by the total number of eligible newly diagnosed HIV-1 cases among MSM in Baotou during the study period.

### Measures and Covariates

Demographic variables were obtained from the National Center for AIDS/Sexually Transmitted Disease Control and Prevention (NCAIDS) information system and included age, ethnicity, marital status, educational level, and registered residence. Clinical variables included cluster of differentiation 4 (CD4+) T cell count at diagnosis. HIV-1 sequence analysis was used to determine viral subtype, identify molecular transmission clustering status (clustered or nonclustered based on genetic distance thresholds), and detect PDR-associated mutations.

### Data Collection and Sources of Secondary Data

Demographic and epidemiological data were obtained from the NCAIDS information system. Laboratory data, including HIV-1 pol gene sequences, were generated using the Illumina next-generation sequencing (NGS) platform.

### HIV-1 Nucleic Acid Extraction, Polymerase Chain Reaction Amplification, and Sequencing

Viral DNA/RNA was extracted using the TGuide S96 Magnetic Bead Method Viral DNA/RNA Extraction Kit (GDP804; TIANGEN Biotech [Beijing] Co, Ltd) according to the manufacturer’s instructions. To study the HIV-1 pol gene region, particularly the first 226 codons within the protease (PR) and reverse transcriptase (RT) regions, triple fragment analysis was performed using one-step RT-polymerase chain reaction (PCR; Takara; RR055A) and nested PCR (Takara; RR901A). Amplification primers and conditions have been previously reported [24]. Electrophoresis (1% agarose gel) confirmed amplicons from PCR-positive specimens. The library was constructed using segmented labeled amplicons, labeled with unique barcode sequences, and mixed according to the results of DNA quantification to ensure that the DNA input of individual samples was ≥50 ng and the total library amount was ≥150 ng. The prepared libraries were sent to the sequencing company to be analyzed by second-generation sequencing using the Illumina High-Throughput Sequencing Platform after passing the quality test by connecting the P5/P7 sequencing connectors at both ends of the DNA fragments.

### Sequence Processing and HIV-1 Subtyping

The second-generation sequencing data in FASTA format provided by the sequencing company were preprocessed under a Linux environment by Mothur software (Patrick D Schloss) for quality control to remove low-quality sequences and mixed sequences. Batch processing in Mothur was performed to generate consensus sequences using a quality-score cutoff of 20. Quality control was performed by the HIV database, excluding sequences with >5% mixed bases and those containing stop codons [25]. The obtained sequence fragments were calibrated and assembled using Sequencer (v5.4.5; GeneCodes). After preliminary processing, the sequences were manually aligned and edited in BioEdit (v7.7.1; Tom Hall, Ibis Biosciences) and compared with reference sequences in the HIV database at Los Alamos National Laboratory. Phylogenetic analysis was subsequently performed in MEGA (v6.0; Koichiro Tamura, Glen Stecher, Daniel Peterson, Alan Filipski, and Sudhir Kumar) using the neighbor-joining method. For circulating recombinant form (CRF), clustering analysis was first conducted with established international reference strains, and additional validation was performed using the COMET (Daniel Struck) online tool [26]. Sequences not clustered with known reference strains were classified as unique recombination forms (URFs) and further analyzed for recombination patterns using the RIP (Siepel AC, Halpern AL, Macken C, Korber BT) [27] and jpHMM (Anne-Kathrin Schultz) [28] online tools.

### HIV Molecular Transmission Network Analysis

To analyze the characteristics of HIV-1 transmission among MSM in Baotou, a molecular network was constructed using collected pol gene sequence data. First, pairwise genetic distances between aligned sequences were calculated using HyPhy (v2.2.4; Sergei L Kosakovsky Pond). Nodes were connected when the pairwise genetic distance between their corresponding sequences fell below the predefined threshold. To more reliably identify infected individuals with potential transmission links within the molecular network, an optimal threshold was selected by maximizing the number of clusters, thereby avoiding network fragmentation or excessive merging. Consequently, sequence pairs with genetic distances <1.30% were used to construct the molecular transmission network. Finally, the network was visualized using Cytoscape (v3.5.1; Cytoscape Consortium). The assortativity coefficient (ra) is an important measure of network assortativity, with values ranging from −1 to 1 [29]. A value close to 1 indicates that the network exhibits high assortativity, meaning similar nodes are more likely to connect with each other. A value close to −1 indicates that the network exhibits high disassortativity, meaning nodes of different types are more likely to connect with each other. A value close to 0 indicates that the network connections are random.

### Drug Resistance Analysis

According to the National Technical Specifications for AIDS Testing, as outlined by the Chinese Center for Disease Control and Prevention, the sequenced data were forwarded to the HIV Drug Resistance Database of Stanford University [30]. Specifically, the HIV database program’s sequence analysis online feature was used to assess the genotypic drug resistance mutations and resistance levels in HIV-1-infected MSM. The clinical significance of drug resistance, encompassing protease inhibitors (PIs), nucleoside reverse transcriptase inhibitors (NRTIs), and nonnucleoside reverse transcriptase inhibitors (NNRTIs), is categorized into 5 distinct levels for each antiretroviral medication: susceptible, possible resistance, low resistance, moderate resistance, and high resistance. Any level of resistance, from low to high, is deemed resistant. The prevalence of drug resistance is calculated as the proportion of individuals with infections resistant to 3 drugs among all newly reported HIV-1 infections.

### Statement on Missing Data

Of the 213 enrolled participants, plasma amplification and sequencing were successful in 176 (82.6%). The 37 cases with missing sequence data did not differ significantly from those with available sequences in terms of age, marital status, education, or baseline CD4+ lymphocyte count (all *P*>.05). For all 176 participants with sequence data, no values were missing for the primary outcomes (clustering and PDR), demographic variables, or covariates. The small proportion of missing sequences was handled by complete-case analysis for the molecular network and logistic regression and by using the total enrolled sample as the denominator for PDR prevalence to provide a conservative estimate.

### Ethical Considerations

The study was approved by the Biomedical Ethics Review Committee of Inner Mongolia Center for Disease Control and Prevention (approval number 202411282). All procedures were conducted in accordance with the Declaration of Helsinki. Written informed consent was obtained from every participant prior to blood sample collection. The consent form explicitly stated that the specimen and associated clinical data would be used for HIV molecular epidemiology research, including genetic sequencing and drug resistance analysis. All study data were deidentified. During sequencing and data analysis, participants were identified solely by unique study codes; personal identifiers were removed before data analysis. Only authorized research team members had access to the key data. No financial compensation was provided to participants, as sample and data collection were carried out as part of routine public health surveillance and clinical care, with no additional study-specific procedures beyond standard post-diagnosis evaluation. No images, photographs, or any other materials that could reveal the identity of individual participants are included in this manuscript or its supplementary files.

### Statistical Analysis

Demographic and baseline characteristics were summarized according to variable type. Categorical variables were expressed as frequencies and percentages, while continuous variables were assessed for normality and presented as medians with IQRs, as appropriate. To identify factors associated with clustering within the molecular transmission network, univariate logistic regression analysis was first performed to examine the association between each independent variable and the outcome variable (clustered vs nonclustered). To control for potential confounding effects, all factors found to be statistically significant (*P*<.05) in the initial univariate analysis were included in a multivariate logistic regression model. A stepwise selection approach was used to construct the final model, retaining only those variables that remained statistically significant (*P*<.05) after controlling for all other factors. Odds ratios (OR) and their corresponding 95% CIs were calculated to estimate the strength of associations. This modeling strategy was chosen because the outcome variable (molecular clustering) was binary, and logistic regression is the appropriate method for identifying independent predictors of binary outcomes in cross-sectional epidemiological data. Network assortativity was assessed using the assortativity coefficient (ra), which measures the tendency of nodes with similar attributes to connect with one another. The coefficient ranges from −1 (disassortative) to 1 (assortative). All data analyses were performed using SPSS (version 25; IBM Corp) software, and figures were generated using GraphPad Prism (version 8; GraphPad Software LLC) and R (version 4.4.2; R Foundation for Statistical Computing) software.

## Results

### Demographic Characteristics of the Study Population

This study involved gathering samples from 213 newly diagnosed HIV-1 cases among MSM, achieving a high sampling rate of 91.42% (n=213). Among newly reported cases, HIV-1 infections accounted for 32.86% (n=70) in 2022, 36.62% (n=78) in 2023, and 30.52% (n=65) in 2024. Of these, we successfully sequenced and analyzed 82.62% (n=176) of the samples. A total of 63 sequences were obtained in 2022, 64 in 2023, and 49 in 2024. As shown in Table 1, among the sequenced participants, the majority were 30‐50 years old (n=91, 51.70%). About 66.48% (n=117) were never-married individuals, and 79.55% (n=140) had at least a senior middle school education. CRF07_BC (n=100, 56.82%) was the most common subtype, followed by CRF01_AE (n=51, 28.98%), CRF55_01B (n=10, 5.68%), B (n=6, 3.41%), and other (n=9, 5.11%).

### Subtype Distribution Characteristics

The MSM population newly reported with HIV-1 infection over the past 3 years includes 7 subtypes and 4 URFs. The subtypes in this region are primarily composed of CRF07_BC (n=100, 56.82%) and CRF01_AE (n=51, 28.98%), followed by CRF55_01B (n=10, 5.68%), B (n=6, 3.41%), and others (n=9, 5.11%), including CRF67_01B (n=3, 33.33%), CRF59_01B (n=1, 11.11%), CRF08_BC (n=1, 11.11%), and URF (n=4, 44.44%; Figure 1). The CRF07_BC-N subclusters dominate in these current circulating strains (Figure 1B). Among never-married individuals under the age of 50 with a high school education or higher, CRF07_BC is a common subtype. In analyzing the demographic characteristics of HIV-1 infection among MSM, no significant association among infection report year, personal age, race, education level, marital status, or registration location and viral infection was found (*P*>.05). There were significant differences in the genotypic composition of HIV-1 MSM infections across different CD4+ T lymphocyte counts (*P*<.05).

**Figure 1.**
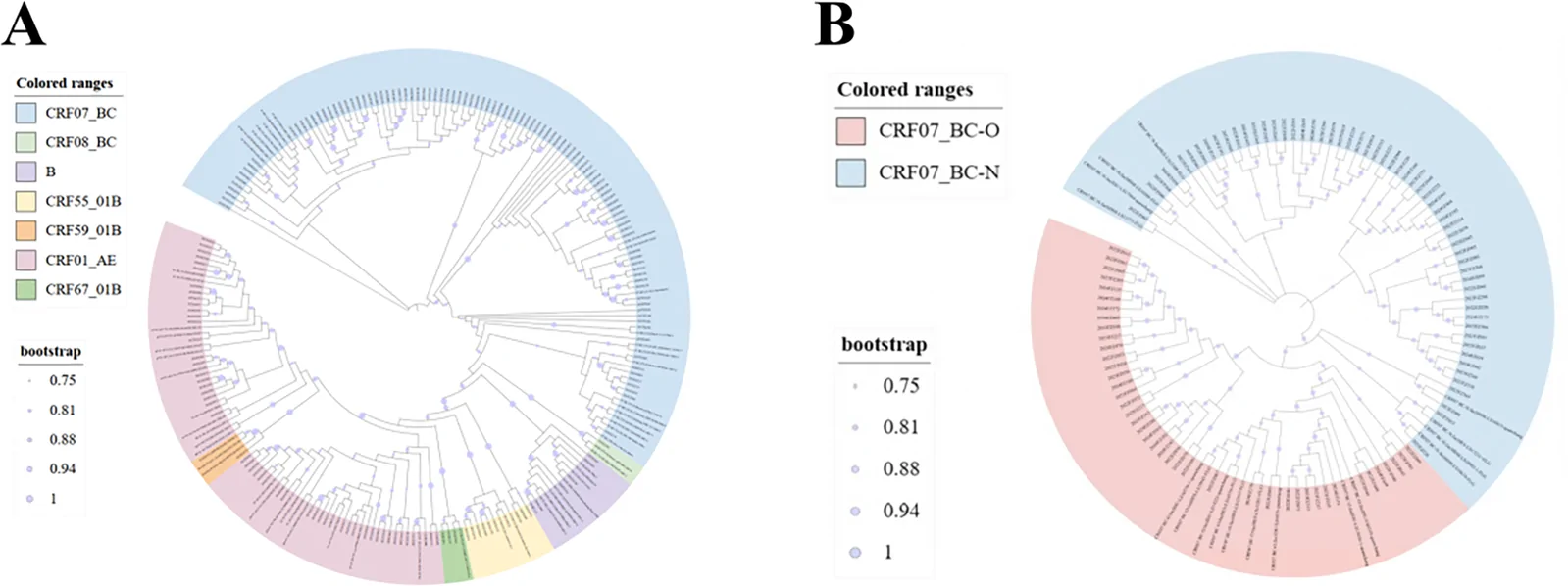
Phylogenetic tree of HIV-1 pol sequences inferred through maximum likelihood. (A) Shows the phylogenetic tree of all subtypes except unique recombination forms (URFs). (B) Shows the phylogenetic tree of CRF07_BC. Different colors represent different subclusters. CRF: circulating recombinant form.

### Molecular Transmission Network Analysis

The number of clusters and the number of nodes included in the network were calculated separately for genetic distances ranging from 0.10% to 2%. The molecular transmission network was constructed using a genetic distance threshold of 1.30%, which yielded the largest number of clusters. Among the 176 pol gene sequences, 52 sequences (29.55%) were included as nodes in the network using a genetic distance threshold of 0.013, distributed across 19 clusters. Cluster sizes ranged from 2 to 6 nodes, 12 out of 19 clusters consisted of only 2 nodes connected by a homosexual transmission link, and 7 out of 19 clusters (36.84%) had 2 or more links. The largest cluster contained 6 nodes (11.54%) and 15 edges (32.61%). The median pairwise genetic distance was 0.0070 (IQR 0.0030‐0.0103). Node degrees ranged from 1 to 5, with most nodes in the network having a degree of 1. Among these 19 clusters, CRF07_BC accounted for 73.68% (n=14), CRF01_AE for 15.79% (n=3), and CRF55_01B for 10.53% (n=2). In addition, we identified 2 small drug resistance clusters in the network, both belonging to the CRF07_BC subtype. The key drug resistance mutations identified in the 2 clusters were Q58E and K103N, respectively.

Then, this study analyzed the assortative coefficients of demographic characteristics to better reflect assortative mixing within molecular transmission networks. The results indicate that the network exhibited significant assortative mixing by age (ra=0.41; *P*=.001), marital status (ra=0.408; *P*=.002), and educational attainment (ra=0.385; *P*=.001), rather than random mixing (Figures 2A-2F). Therefore, we analyzed the connection strength for these 3 demographic characteristics (Figures 3A-3C). The visualization results show that, regarding age, the connection strength was highest among older individuals (>50 years; n=14, 31.11%). The connection strength was higher among married individuals (n=17, 37.78%). Nodes with a senior high school education and above were more likely to establish connections (n=26, 57.78%).

**Figure 2.**
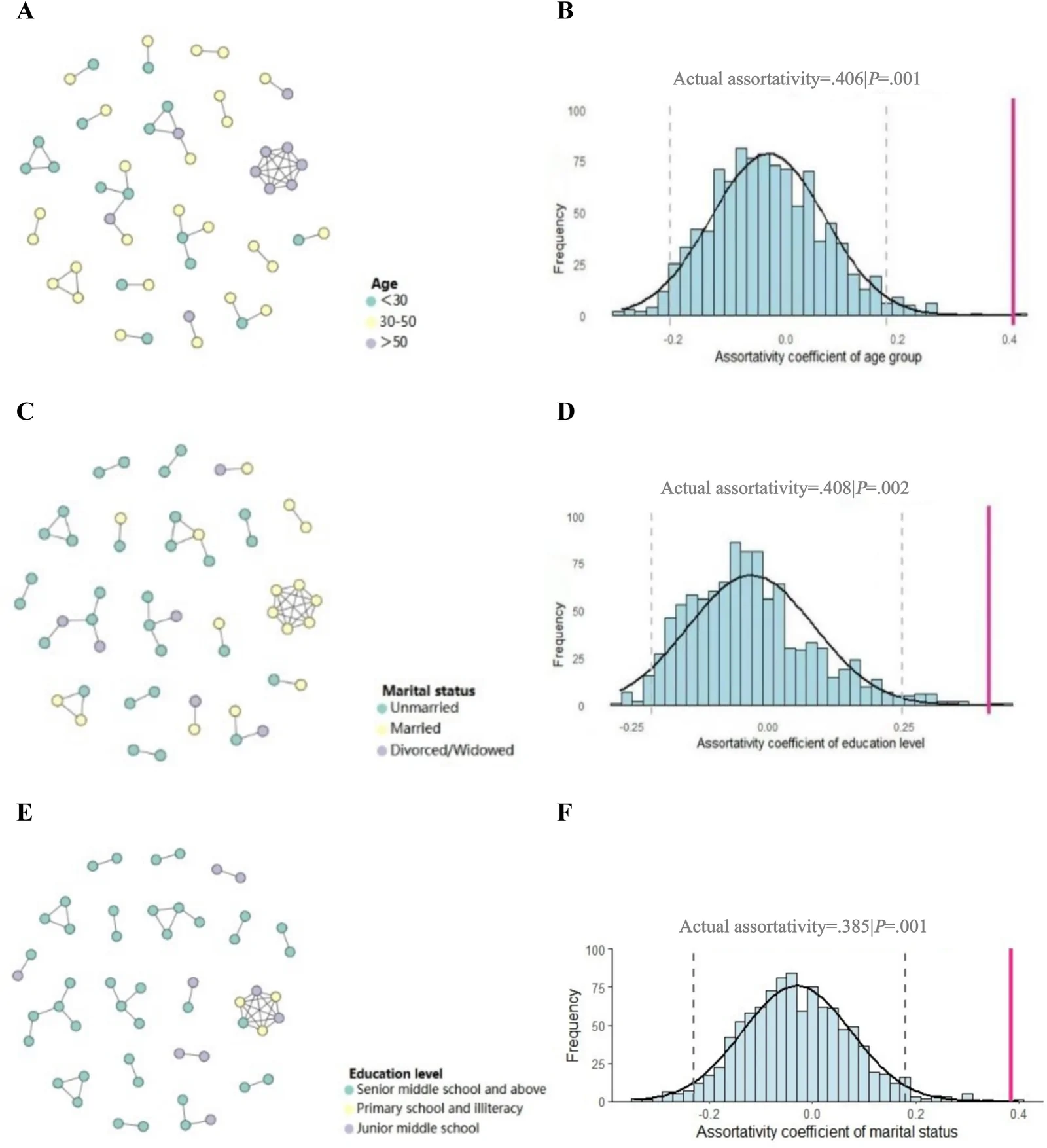
Assortativity within the HIV-1 molecular transmission network. (A) Clusters of HIV-1 molecules coded by age group. (B) Assortativity analyzed by age group. (C) Clusters of HIV-1 molecules coded by marital status group. (D) Assortativity analyzed by marital status group. (E) Clusters of HIV-1 molecules coded by education level. (F) Assortativity analyzed by education level. The gray dashed line represents the 5% significance level. Pink vertical lines represent the actual assortativity. A value close to 1 indicates that the network exhibits high assortativity, meaning similar nodes are more likely to connect with each other.

**Figure 3.**
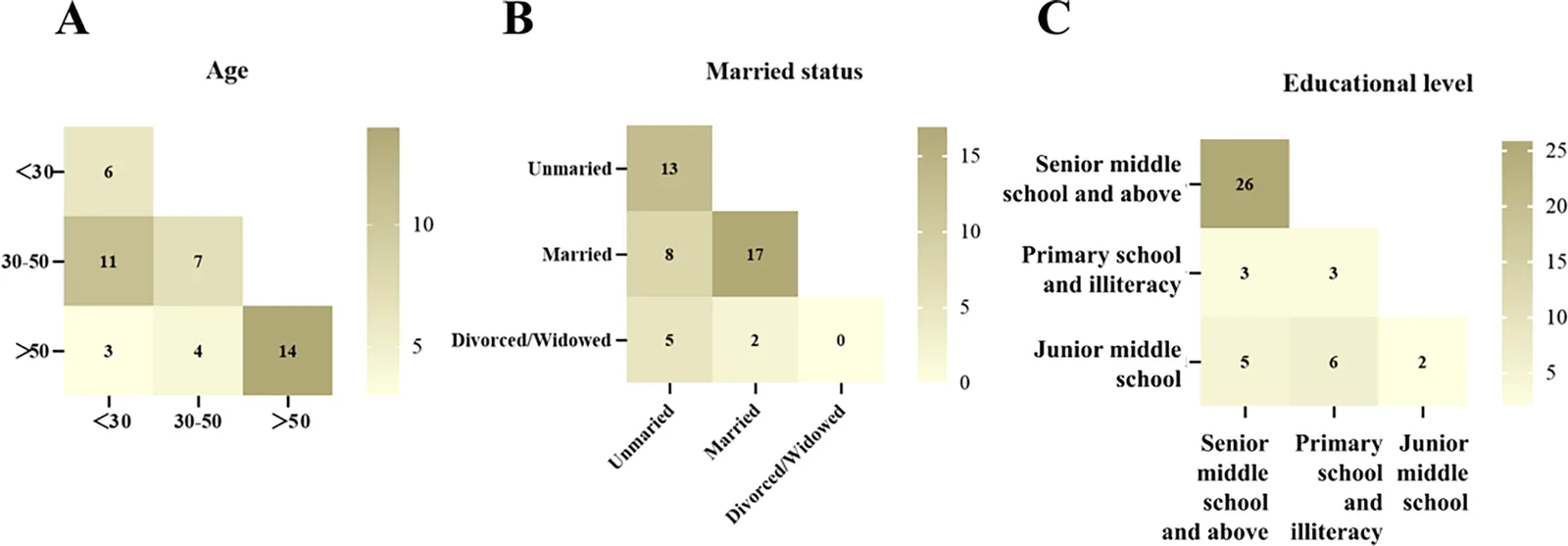
The number of connections between nodes in a network under 3 different characteristics. (A) Age. (B) Marital status. (C) Education level. The numbers in the cross-grid units represent the number of correlations between HIV-1 individuals in the 2 groups.

### Factors Associated With Clustering

Results from multivariate logistic regression indicated that, among MSM, marital status and subtype were most strongly associated with clusters of infections (Table 2). Compared with CRF01_AE, CRF07_BC was more likely to be associated with clusters of infections (OR 4.98; 95% CI 1.92‐12.94). Furthermore, compared with never-married individuals, those who were divorced or widowed were less likely to experience clustering (OR 0.27, 95% CI 0.08‐0.92). Other factors, such as age, year of reporting, educational level, ethnicity, pretreatment CD4+ T cell count, presence of PDR, and region of registration, were not associated with clustering (Table 2).

**Table 2.** Analysis of risk factors for HIV-1 infection clusters among men who have sex with men (MSM).

Variables	Number, n(%)	Clustered, n (%)	*P* value	Crude OR^a^ (95% CI)	*P* value	Adjusted OR (95% CI)
Reported year, n (%)						
2022	63 (35.80)	21 (33.33)	—^b^	Reference	—	
2023	64 (36.36)	18 (28.13)	.52	0.64 (0.67‐1.52)	—	—
2024	49 (27.84)	13 (26.53)	.44	0.66 (0.26‐1.60)	—	—
Subtype, n (%)						
CRF^c^01_AE	51 (28.98)	6 (11.76)		Reference		Reference
CRF07_BC	100 (56.82)	41 (41.00)	<.001	5.21 (2.04‐13.34)	<.001	4.98 (1.92‐12.94)
CRF55_01B	25 (14.20)	5 (20.00)	.06	2.78 (0.96‐8.01)	.06	2.86 (0.97‐8.44)
Age (years), n (%)						
<30	59 (33.52)	16 (27.12)		Reference	—	—
30‐50	91 (51.70)	26 (28.57)	.07	0.88 (0.42‐1.82)	—	—
>50	26 (14.77)	10 (38.46)	.48	0.70 (0.26‐1.90)	—	—
Ethnicity, n (%)						
Han Chinese	162 (92.05)	48 (29.63)	—	Reference	—	—
Mongol	12 (6.82)	3 (25.00)	.73	1.26 (0.32-4.87)	—	—
Hui	2 (1.14)	1 (50.00)	.54	0.42 (0.02-6.87)	—	—
Registered residence, n (%)						
Baotou	155 (88.07)	47 (30.32)	—	Reference	—	—
Other regions	16 (9.09)	4 (25.00)	.66	1.30 (0.40-4.26)	—	—
Other provinces	5 (2.84)	1 (20.00)	.62	1.74 (0.18-15.99)	—	—
Marital status, n (%)						
Never married	117 (66.48)	29 (24.79)	—	Reference	—	Reference
Married	34 (19.32)	17 (50.00)	.01	0.34 (0.16-0.76)	.61	0.75 (0.24-2.26)
Divorced/widowed	25 (14.20)	6 (24.00)	.55	1.38 (0.48-4.00)	.04	0.269 (0.08-0.92)
Education level, n (%)						
Primary school and illiteracy	10 (5.68)	3 (33.33)	—	Reference	—	—
Junior middle school	26 (14.77)	9 (34.62)	.96	0.96 (0.20-4.72)	—	—
Senior middle school and above	140 (79.55)	40 (28.57)	.96	1.04 (0.26-4.20)	—	—
Occupation, n (%)						
Unemployment	74 (42.05)	19 (25.68)		Reference	—	—
Farmers and herders	17 (9.66)	9 (52.94)	.03	0.31 (0.10-0.91)	—	—
Students	13 (7.39)	2 (15.38)	.34	1.90 (0.38-9.36)	—	—
Business service	19 (10.80)	6 (31.58)	.60	0.74 (0.24-2.24)	—	—
others	53 (30.11)	16 (30.19)	.58	0.80 (0.36-1.75)	—	—
CD4+^d^ T cell count, n (%)						
<200	43 (24.43)	13 (30.23)	—	Reference	—	—
200‐500	91 (51.70)	29 (31.87)	.56	0.78 (0.36-1.74)	—	—
>500	42 (23.86)	10 (23.81)	.67	1.24 (0.468-3.28)	—	—
Drug resistance, n (%)						
Yes	14 (7.95)	6 (42.86)	—	Reference	—	—
No	162 (92.05)	46 (28.40)	.26	1.89 (0.62-5.75)	—	—

a OR: odds ratio.

b Not available.

c CRF: circulating recombinant form.

d CD4+: cluster of differentiation 4.

### Drug Resistance in HIV‐1‐Infected MSM

The prevalence of PDR increased from 4.29% (n=3) in 2022 to 9.23% (n=6) in 2024. The overall prevalence of PDR in the HIV-1-infected MSM population was 6.57% (n=14). A chi-square test for trend revealed a significant increasing trend in PDR prevalence over the 3-year period (*χ*^2^_1_=17.885; *P*<.001), showing an increasing trend in PDR. NNRTI resistance was the most common (n=8, 3.76%), followed by PI resistance (n=5, 2.35%), and NRTI resistance (n=3, 1.14%).

For HIV-1-infected MSM in Baotou, NNRTIs were associated with the most common resistance mutations, with the K103N mutation (n=5, 2.35%) being the most frequent, followed by L100I (n=2, 0.94%). For NRTIs resistance, the most frequently detected mutation was D67N/del (n=2, 0.94%), while for PIs resistance, Q58E (n=3, 1.41%) was the most common mutation.

As shown in Table 3, mutations associated with NNRTIs primarily result in moderate/high resistance to doravirine (DOR), efavirenz (EFV), etravirine (ETR), nevirapine (NVP), and rilpivirine (RPV), with 5 cases exhibiting high resistance to EFV and 7 cases exhibiting high resistance to NVP. Mutations associated with PIs primarily result in low resistance to nelfinavir (NFV) and tipranavir boosted with ritonavir (TPV/r). Mutations associated with NRTIs primarily result in moderate resistance to abacavir (ABC), zidovudine (AZT), lamivudine (3TC), emtricitabine (FTC), and tenofovir disoproxil fumarate (TDF), and high resistance to stavudine (D4T) and didanosine (DDI). In addition, this study used chi-square tests to assess the association between subtypes and the presence of resistance. Significant differences were observed, with resistance more likely to be found in the CRF07_BC subtype (*χ*^2^_8_=90.44; *P<*.001).

**Table 3.** Distribution of drug susceptibility and resistance mutations in HIV-1-infected men who have sex with men (MSM).

Drugs		Low-Level resistance		Intermediate resistance		High-Level resistance	
		Numbers	Mutations	Numbers	Mutations	Numbers	Mutations
PIs^a^							
NFV^b^		1	N83D	1	M46I	—^c^	—
TPV/r^d^		4	Q58E	—	—	—	—
NRTIs^e^							
ABC^f^		2	T215Y	1	D67del+S86del	—	—
AZT^g^		1	D67N+K70N	1	T215Y	—	—
D4T^h^		—	—	1	D67n+K70N	1	D67del+S86del
DDI^i^		1	D67N+K70N	—	—	1	D67del+S86del
3TC^j^		—	—	1	D67del+S86del	—	—
FTC^k^		—	—	1	D67del+S86del	—	—
TDF^l^		1	D67N+K70N	1	D67del+S86del	—	—
NNRTIs^m^							
DOR^n^		2	L100I	1	K103N,P225H	1	F227L
EFV^o^		1	F227L	—	—	7	K103N,P225H,L100I
ETR^p^		—	—	2	L100I	—	—
NVP^q^		—	—	1	F227L	7	K103N,P225H,L100I
RPV^r^		—	—	—	—	2	L100I

a PI: protease inhibitor.

b NFV: nelfinavir.

c Not applicable.

d TPV/r: tipranavir boosted with ritonavir.

e NRTI: nucleoside reverse transcriptase inhibitor.

f ABC: abacavir.

g AZT: zidovudine.

h D4T: stavudine.

i DDI: didanosine.

j 3TC: lamivudine.

k FTC: emtricitabine.

l TDF: tenofovir disoproxil fumarate.

m NNRTI: nonnucleoside reverse transcriptase inhibitor.

n DOR: doravirine.

o EFV: efavirenz.

p ETR: etravirine.

q NVP: nevirapine.

r RPV: rilpivirine.

## Discussion

### Principal Findings

China’s economic development is characterized by substantial regional disparities. Western regions are geographically vast, relatively underdeveloped economically, and feature widely dispersed populations, which pose considerable challenges for disease prevention and control. Targeted interventions based on HIV-1 molecular transmission networks have been widely implemented in economically developed regions and have demonstrated promising effectiveness in HIV prevention and control. Meanwhile, with the continuous advancement of NGS technologies, these approaches have also been increasingly applied to HIV drug resistance surveillance, thereby providing improved guidance for ART.

The Inner Mongolia Autonomous Region, located in northern China’s border area, remains relatively underdeveloped economically and has limited experience in the application of novel HIV prevention technologies and methodologies. Therefore, this study selected Baotou, Inner Mongolia, as the study site and applied HIV-1 molecular transmission network analysis combined with NGS technology to investigate transmission clustering and pretreatment drug resistance among local HIV-infected individuals. With a sampling rate exceeding 90%, the study is representative of this population. The findings are expected to provide more precise and efficient strategies for HIV prevention and control in Baotou and potentially across the entire Inner Mongolia Autonomous Region.

Demographic analysis indicates that HIV-1-infected MSM in Baotou tend to be younger, never-married, and better educated. Similar results have been reported in other studies on MSM populations [31,32]. Previous studies have shown that young MSM in the Inner Mongolia Autonomous Region have a high level of HIV/AIDS knowledge. However, the proportion of commercial sex has increased annually, and the rate of consistent condom use remains low, resulting in a disconnect between knowledge and behavior. This may explain the relatively high infection rate among young MSM in the region [33]. For MSM engaging in unprotected receptive anal sex, interventions that reduce infection risk are critical. Therefore, this group should be a priority for prevention and control efforts, with screening expanded to include both infected individuals and their sexual partners.

Among HIV-1-infected MSM in Baotou, the predominant subtypes are CRF07_BC and CRF01_AE. Phylogenetic analysis indicates that the CRF07_BC-N subclusters are the dominant epidemiological cluster. CRF07_BC-N subclusters originated in Yunnan Province and are primarily transmitted among MSM [34]. Compared with the CRF07_BC-O subclusters, CRF07_BC-N subclusters exhibit higher network density, interprovincial transmission rates, and interregional transmission rates [35]. The results of this study further indicate that this lineage may have undergone large-scale interprovincial and intraprovincial transmission across China. Therefore, strengthening the diagnosis and surveillance of HIV-1-infected MSM is crucial for controlling the spread of CRF07_BC. With the increase in homosexual transmission, CRF01_AE has become one of the predominant strains among MSM [36,37]. In Hebei Province [38] and Heilongjiang Province [39], which border the Inner Mongolia Autonomous Region, the proportion of CRF01_AE among MSM is the highest, indicating that the predominant circulating subtypes among MSM exhibit regional characteristics. Recent studies have identified novel recombinant strains (CRF01_AE/CRF07_BC) among MSM in Beijing [40]. The high frequency of recombination between these 2 subtypes underscores the importance of closely monitoring viral transmission and evolution in high-risk areas and populations. Therefore, continuous surveillance of HIV-1 subtype diversity and transmission dynamics is essential for effective epidemic control.

Molecular network analysis has revealed the transmission patterns of HIV-1 among MSM. Compared to individuals infected with the CRF01_AE strain, those infected with the CRF07_BC strain showed a stronger association with clustered HIV-1 transmission within the network. These findings are consistent with those observed in Chongqing [41] and Guangxi [42]. Pathogenic studies of the CRF07_BC strain indicate a relatively low baseline viral load. A 7-amino acid deletion (Δ7) in the p6gag region impairs viral maturation and release, leading to reduced replicative capacity [43]. Because of its transmission advantages, CRF07_BC may continue to play an important role in HIV transmission among MSM populations in China [44]. Compared with MSM who have never been married, divorced, or widowed, MSM are associated with a lower risk of forming transmission clusters. MSM who have never been married are typically younger and may engage in high-risk sexual behavior, such as frequently changing sexual partners and having unprotected sex. A greater number of sexual partners may increase opportunities for HIV transmission, which in turn may facilitate viral clustering and spread [44]. Although this group has a relatively high level of education, previous studies have shown that there is a discrepancy between their knowledge of HIV/AIDS and their actual behavior in the Inner Mongolia Autonomous Region [33]. Our findings merely indicate that, among HIV-1-infected individuals in Baotou, divorced or widowed MSM are less strongly associated with viral clustering and transmission compared to MSM who have never been married. To confirm these results, further validation through expanded sampling and epidemiological investigations is required.

In this study, the assortative coefficients for age group, marital status, and educational level were statistically significant. HIV-1-infected individuals within the same age group, marital status, and educational level tended to cluster within the network. The connection patterns indicated that older individuals, married individuals, and those with higher educational levels were more likely to connect with similar nodes within the network. Analyzing the assortative characteristics of nodes within a network can rapidly identify key nodes [45]. Therefore, during epidemic surveillance, it is necessary to integrate network indicators with molecular transmission networks to provide critical information on priority populations for population-based epidemic prevention and control, formulate interventions targeting HIV transmission hotspots, and use public health resources more efficiently to achieve precise HIV prevention and control [46].

This study assessed the prevalence of PDR and the presence of drug resistance mutations (DRMs) among MSM in Baotou over the past 3 years. The local PDR rate in Baotou was 6.57%, slightly lower than the national average (7.4% in 2022) [47]. This difference may be attributed to the more stable demographic structure of the MSM population in Baotou, which reduces the likelihood of cross-regional transmission of drug-resistant strains. Despite being slightly below the national average, the local PDR rate still exceeds the World Health Organization (WHO) warning threshold of 5%. This study observed the highest frequency of DRMs among NNRTIs, followed by NRTIs, with resistance of the CRF07_BC subtype being the predominant type. This is consistent with findings reported in certain cities [19,21]. This finding may be associated with the relatively low genetic barrier of NNRTIs and the widespread use of first-line treatment regimens [48-50]. High levels of resistance were primarily observed for drugs such as EFV, NVP, and D4T, highlighting the potential impact of long-term use of the same antiviral treatment regimen and urging the exploration of effective alternative regimens [51,52]. The PDR rate for NNRTIs was 3.76%, higher than that for NRTIs and PIs. Studies indicate that the increase in PDR prevalence is primarily driven by NNRTI resistance [53]. Additionally, it is worth noting that the proportion of dual resistance to NRTIs and NNRTIs among individuals with drug resistance is 14.29%, which limits the options for second-line treatment regimens. This suggests that resistance testing should be conducted on patients with virological failure before considering second-line treatment regimens.

Then this study analyzed the distribution of DRMs. First, the most commonly detected NNRTI-associated resistance mutation is K103N. Multiple studies have demonstrated that K103 is the most common DRM site for NNRTIs used as first-line antiviral drugs, and this mutation is closely associated with resistance to EFV and NVP, and it is also commonly found in CRF07_BC [54-57]. Our research results also prove this point. Therefore, particular vigilance is required for the K103N mutation. The next most common DRM is L100I, which can lead to moderate to high resistance to NVP, RPV, and DPV. We found that several drug-resistant individuals exhibited varying levels of resistance to DOR. A Dutch study demonstrated that DOR holds promise for more effective and cost-effective treatment for patients living with HIV [58], but the results indicate that close monitoring will still be necessary when using DOR in the future.

Our research team recently published a comprehensive analysis of sexually acquired HIV-1 cases in Baotou, which identified older heterosexual men as key drivers of local transmission networks [23]. The present study is a logical extension of that work, specifically designed to conduct an in-depth, dedicated molecular investigation of the MSM population—the group accounting for the highest proportion of newly reported HIV-1 cases in Inner Mongolia. While the two studies share the same geographical setting and methodological framework, they address fundamentally distinct research questions and target populations, and are therefore complementary rather than redundant. This study demonstrates that MSM remain the core population sustaining the epidemic and require dedicated, population-specific attention. Together, these two studies provide a more complete picture of the HIV-1 epidemic in Baotou, with the current manuscript filling a critical gap by delivering the granular, population-specific data necessary for designing targeted interventions for MSM in Inner Mongolia.

### Limitations

This study has certain limitations. First, a portion of the samples was collected during the COVID-19 pandemic, and changes in epidemic prevention measures and health care–seeking behaviors may have resulted in incomplete sampling. Some transmission chains may not have been effectively identified because infected individuals failed to seek medical care, undergo testing, or be included in the surveillance system in a timely manner. This resulted in an incomplete description of the local HIV transmission network and may have underestimated the size of certain transmission clusters. Future studies could incorporate samples and data from multiple time periods to validate these results and reduce the impact of sampling bias on the identification of transmission chains. Second, the proportion of potentially nondisclosed MSM (pnMSM) in the sample may have been underestimated, and reporting bias may have existed during epidemiological data collection, which could introduce bias in cluster analysis and pre-treatment resistance rates. Future studies should include pnMSM to reduce potential reporting bias. Finally, testing for integrase strand transfer inhibitors (INSTI)–associated resistance has not yet been included. Although the reported rate of INSTI-associated resistance is currently low, the potential risk of resistance cannot be ignored given the expanding use of these drugs. Future studies will focus on conducting resistance analyses for these drugs.

### Conclusion

Unlike previous studies that primarily focused on high-burden southeastern and coastal regions of China, this study addresses an important geographical gap by investigating HIV-1 molecular transmission clusters and pretreatment drug resistance among MSM in Baotou, a major city in northern China.

This study is distinguished by its use of NGS technology to provide a comprehensive characterization of the local HIV epidemic by integrating molecular transmission network analysis, assortative mixing analysis, and pretreatment drug resistance surveillance. These findings demonstrate that the high clustering of CRF07_BC and the predominance of NNRTI resistance are not confined to coastal regions with severe HIV epidemics but are also present in northern border regions characterized by different demographic and behavioral patterns.

These results highlight the importance of incorporating molecular network surveillance into routine HIV monitoring systems to identify high-risk transmission clusters and support precision public health interventions. In particular, younger, unmarried, and highly educated MSM exhibited a greater likelihood of being HIV-1-infected and should therefore be prioritized for targeted prevention strategies. Furthermore, surveillance of pretreatment drug resistance may facilitate the optimization of region-specific first-line antiretroviral regimens and improve the efficient allocation of limited health care resources.

In the Inner Mongolia Autonomous Region, the geographically dispersed population and relatively limited health care resources have hindered the widespread implementation of advanced HIV surveillance technologies and targeted intervention strategies. These findings may provide important evidence for molecular surveillance-based HIV prevention strategies in other resource-limited border regions of China.

## Supplementary material

10.2196/82208 Multimedia Appendix 1 Participant flow diagram.

## Abbreviations

3TC: lamivudine
ABC: abacavir
AOR: adjusted odds ratio
ART: antiretroviral therapy
AZT: zidovudine
CD4+: cluster of differentiation 4
CDC: US Centers for Disease Control and Prevention
CRFs: Circulating Recombinant Forms
D4T: stavudine
DDI: didanosine
DOR: doravirine
DRM: drug resistance mutation
EFV: efavirenz
ETR: etravirine
FTC: emtricitabine
INSTI: integrase strand transfer inhibitor
MSM: men who sex with men
NCAIDS: National Center for AIDS/Sexually Transmitted Disease Control and Prevention
NFV: nelfinavir
NGS: next-generation sequencing
NNRTI: nonnucleoside reverse transcriptase inhibitor
NRTIs: nucleoside reverse transcriptase inhibitors
NVP: nevirapine
OR: odds ratio
PCR: polymerase chain reaction
PDR: pretreatment HIV drug resistance
PI: protease inhibitor
pnMSM: potential nondisclosed men who have sex with men
PR: protease
RPV: rilpivirine
RT: reverse transcriptase
TDF: tenofovir disoproxil fumarate
TPV/r: tipranavir boosted with ritonavir
URF: unique recombination form
WHO: World Health Organization
